# Prediction of Missing Flow Records Using Multilayer Perceptron and Coactive Neurofuzzy Inference System

**DOI:** 10.1155/2013/584516

**Published:** 2013-12-17

**Authors:** Samkele S. Tfwala, Yu-Min Wang, Yu-Chieh Lin

**Affiliations:** Department of Civil Engineering, National Pingtung University of Science and Technology, Neipu Hsiang, Pingtung 91201, Taiwan

## Abstract

Hydrological data are often missing due to natural disasters, improper operation, limited equipment life, and other factors, which limit hydrological analysis. Therefore, missing data recovery is an essential process in hydrology. This paper investigates the accuracy of artificial neural networks (ANN) in estimating missing flow records. The purpose is to develop and apply neural networks models to estimate missing flow records in a station when data from adjacent stations is available. Multilayer perceptron neural networks model (MLP) and coactive neurofuzzy inference system model (CANFISM) are used to estimate daily flow records for Li-Lin station using daily flow data for the period 1997 to 2009 from three adjacent stations (Nan-Feng, Lao-Nung and San-Lin) in southern Taiwan. The performance of MLP is slightly better than CANFISM, having *R* ^2^ of 0.98 and 0.97, respectively. We conclude that accurate estimations of missing flow records under the complex hydrological conditions of Taiwan could be attained by intelligent methods such as MLP and CANFISM.

## 1. Introduction

Taiwan is situated on typhoon tracks with high temperatures and heavy rainfalls. There are over 350 typhoons and about 1000 storms that have attacked Taiwan over the past century and led to severe flood disasters. These events concentrate in the summer and autumn season (June to August), resulting in average annual precipitation of about 2500 mm and reaches 3000–5000 mm in the mountain regions. In addition, rivers in Taiwan are short with small drainage basins and steep slopes. During the above-said period, their peak flows are enormous; for example, a catchment area of about 2000–3000 km^2^ often receives peak flows of up to 10000 m^3^/s [1]. Consequently, measurement instruments installed in some stations are damaged resulting in data gaps. Field personnel may also attribute the data gaps to a number of factors such as malfunctioning of monitoring instrument, absence of observer, natural phenomena (e.g., earthquakes and landslides), and human induced factors like mishandling of observed records. These gaps and discontinuities lead to problems in planning of water development schemes, design of hydraulic structures, and management of water resources. In addition, challenges in the future may surface when a modelling system or a decision support system requires making use of this measured data. This necessitates filling the gaps.

Regression techniques have long been used for the generation of stream flow [2]. The idea is to model flow at one gauge as a function of flow at another gauge or gauges. Reference [3] compared regression and time-series techniques to synthesize and predict stream flow at downstream gauge from an upstream gauge in California. Reference [4] successfully filled in missing data, by extending single-output box-Jenkins transfer/noise models for several groundwater head series to a multiple-output transfer/noise models. However, such methods may not be suitable in Taiwan because of the complex hydrological system.

Artificial neural networks (ANN) are gaining popularity, especially over the last few years, in terms of hydrological applications. At the beginning early nineties, it has been successfully applied in hydrology related areas such as rainfall-runoff modelling [5, 6], stream flow forecasting [7, 8], ground water modelling [9], and reservoir operations and modelling [1, 10]. Reference [11] applied ANN and adaptive neurofuzzy inference system (ANFIS) models to model and predict precipitation 12 months in advance. Reference [12] employed a distributed support vector regression model (D-SVR) equipped with genetic algorithm based artificial neural network (ANN-GA) as part of flood control measures. ANN has also been used successfully in water quality, water management policy, evapotranspiration, precipitation forecasting, and hydrological time series. Most hydrological processes exhibit temporal and spatial variability and are often plagued by issues of nonlinearity of physical processes and uncertainty in spatial estimates. The time and effort required in developing and implementing such complicated models may not be justified. Simpler neural network forecast may therefore seem attractive as an alternative tool.

Reference [13] compared six different types of ANN, namely, the multilayer perceptron network and its variation (the time lagged feed forward network), the radial basis function, recurrent neural network and its variation (the time delay recurrent neural network), and the counter propagation fuzzy neural network for infilling missing daily total precipitation. The results of their experiment revealed that the multilayer perceptron network could provide the most accurate estimates of the missing precipitation. In recent years, much attention has been given to derive effective data driven neurofuzzy models due to its numerous advantages [14]. Reference [15] modeled inflow forecasting of the Nile River using neurofuzzy model. Reference [16] applied neurofuzzy model for evapotranspiration modelling.

To the knowledge of the authors, no work has been reported in the literature that investigates the accuracy of multilayer perceptron (MLP) neural networks model and coactive neurofuzzy inference system model (CANFISM) in missing flow records. Hence, in this study, MLP and CANFISM are used to estimate daily flow records for Li-Lin station using daily flow data for the period 1997 to 2009 from three adjacent stations (Nan-Feng, Lao-Nung, and San-Lin). The above stations are located in the Kaoping river basin in southern Taiwan.

## 2. Materials and Methods

### 2.1. Study Area Characteristic

**Figure 1:**
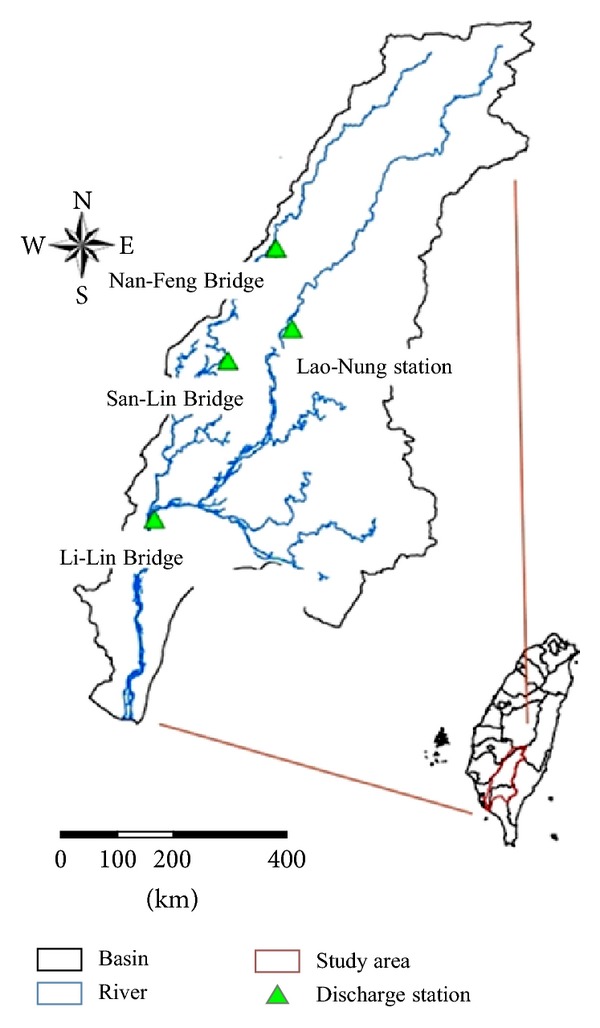
Location of the study area.

Kaoping River basin is located in the southern part of Taiwan at 22°12′30′′ North latitude and 120°12′0′′ East longitude and is shown in Figure 1. In this basin, four flow observation stations were selected and these are Nan-Feng Bridge, San-Lin Bridge, Lao-Nung, and Li-Lin Bridge. This river basin is the largest and most intensively used basin in Taiwan. It is Taiwan's second longest river with its 171 km length and drains a catchment covering 3,257 km^2^ of land that is roughly 9% of the island's total area.

### 2.2. Neural Networks Model

An ANN is an information-processing paradigm inspired by biological nervous systems such as our brain [17]. Neural networks are composed of neurons as basic units. Each neuron receives input data, processes the input data, and transforms them into output forms. The input may be pure data or the output results of other neurons and the output forms may be the results of other neurons [18]. The neural networks used in the study (MLP and CANFISM) are managed by the Neurosolution software version 5.07 presented by the Neurodimension and further descriptions are given below.

#### 2.2.1. Multilayer Perceptron Neural Network

An MLP distinguishes itself by the presence of one or more hidden layers, with computation nodes called hidden neurons, whose function is to intervene between the external inputs and the network output in a useful manner. By adding hidden layers, the network is enabled to extract higher order statistics. The network acquires a global perspective despite its local connectivity due to the extra synaptic connections and the extra dimension of neural network interconnections. The MLP can have more than one hidden layer; however, studies have revealed that a single hidden layer is enough for ANN to approximate any complex nonlinear function [19, 20]. Therefore, in this study, one hidden layer MLP is used. MLP is trained using the many kinds of backpropagation algorithm.

The training performance is a process of adjusting the connection weights and biases so that its output can match the desired output best. Specifically, at each setting of the connection weights, it is possible to calculate the error committed by the networks by taking the difference between the desired and actual responses [21, 22]. In this study, we use Quickprop backpropagation algorithm (BPA). The advantage of this algorithm is that it operates much faster in the batch mode than conventional BPA. In addition, it is not sensitive to the learning rate and the momentum [22]. Throughout all the simulations, the numbers of hidden layer neurons (PE) were found using trial and error method. In total, there were 1283 patterns of data from which 70% was used for training, 20% for cross validation, and 10% used for testing.

**Figure 2:**
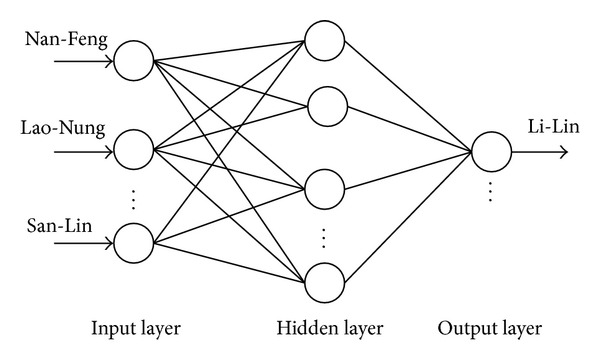
Architecture of the MLP model.

Table 1 shows the condition of the training performance variables for the MLP and Figure 2 shows the developed structure of MLP with 3 inputs of the 3 adjacent stations (Figure 1) from which missing flow records are estimated. The training performance of neural network is iterated until the training error is attained to the training tolerance. Iteration refers to a one completely pass through a set of inputs and target data.

#### 2.2.2. Coactive Neurofuzzy Inference System Model

Coactive neurofuzzy inference system model (CANFISM) belongs to a more general class of adaptive neurofuzzy inference system model (ANFISM). It may be used as a universal approximator of any nonlinear function. In addition, it integrates adaptable fuzzy inputs with a modular neural network to rapidly and accurately approximate complex functions. The characteristics of CANFISM are emphasized by the advantages of integrating neural networks with fuzzy inference in the same topology. The powerful capability of CANFISM stems from pattern-dependant weights between the consequent layer and the fuzzy association layer [23]. The fundamental component of CANFISM is a fuzzy node that applies membership functions to the input nodes. Two membership functions commonly used are general bell and Gaussian. The network also contains a normalization axon to expand the output into a range of 0-1. The second major component of this type of CANFISM is a modular network that applies functional rules to the inputs. The number of modular networks matches the number of network outputs and the number of processing elements in each network corresponds to the number of membership functions. CANFISM also has a combiner layer that applies the membership functions outputs to the modular network outputs.  Table 2 shows the conditions of the training performance variables of the CANFISM.

**Figure 3:**
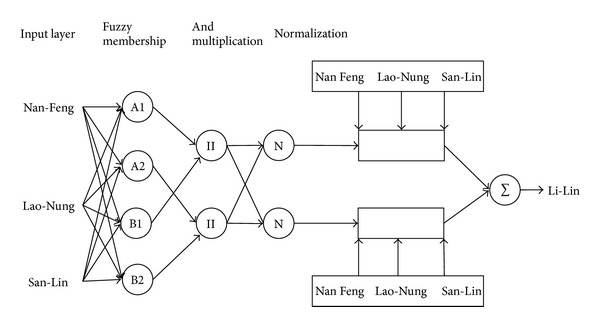
Architecture of the CANFISM model.

In this study, the CANFISM architecture used had three inputs and one output. The flow data from Nan-Feng Bridge, San-Lin Bridge, and Lao-Nung were used as inputs to the model and Li-Lin Bridge as output (Figure 3). From the 1283 patterns of data, 70% of the data were used for training, 20% for cross validation, and 10% for testing the CANFISM model. From the two available membership functions in the model (Bell and Gaussian), the membership function used in this study which is the Gaussian fuzzy axon type which uses a Gaussian shaped curve as its membership function to each neuron. The advantage of this function is that the fuzzy synapses help in characterizing inputs that are not easily discretized [13]. The number of membership functions assigned to each network input was varied between 1 and 10. In the various algorithms (i.e., Levenberg-Marquardt, Delta-Bar-Delta, Step, Momentum, Conjugate Gradient, and Quickprop), we used Quickprop due to the various advantages stated by [21]. Besides, different transfer functions (i.e., Sigmoid, Linear Sigmoid, Tanh, Linear Tanh, Linear, and Bias) were used to identify the one that gives the best results in depicting the nonlinearity of the modeled natural system. The best network architecture for each function was determined by trial and error and was selected based on the one that resulted in minimum errors and best correlation.

### 2.3. Data Normalization

Preprocessing of the data is usually required before presenting the data samples to the neural network [6]. Hence, stream flow data of the stations used were normalized to prevent problems associated with extreme values. In this study, the data is scaled in the range (0-1) using the following equation:

(1) $$\begin{array}{c} Y_{\mathrm{norm}}=\frac{Y_{i}-Y_{\min}}{Y_{\max}-Y_{\min}}, \end{array}$$

where *Y*_norm⁡_ is the scaled input value, *Y*_*i*_ is the actual unscaled observed flow input, and *Y*_min⁡_ and *Y*_max⁡_ refer to the minimum and maximum values of the data, respectively. In addition, some of the data were similar for some days in the different stations; these data was assumed incorrect, and therefore we discarded it.

### 2.4. Models Performance Evaluation

The performance of the neural networks models are evaluated using a variety of standard statistical indexes. In our study, we evaluated the models using three indexes, root mean square error (RMSE), mean absolute error (MAE), and coefficient of correlation (*R*). The RMSE is a measure of the residual variance. MAE measures how close forecasts or predictions are to eventual outcomes. The *R* is a measure of accuracy of a hydrological modelling and is generally used for comparison of alternative models

(2) $$\begin{array}{c} \text{RMSE}=\sqrt{\frac{\sum_{i=1}^{N}{\left(y_{i}-y_{i}^{\prime }\right)}^{2}}{N}},\\ \text{MAE}=\frac{1}{N}\sum_{i=1}^{N}\left|y_{i}-y_{i}^{\prime }\right|,\\ r=\frac{\sum_{i=1}^{N}\left(y_{i}-\overset{-}{y}\right)\left(y_{i}^{\prime }-\overset{-}{y}\right)}{\sqrt{\sum_{i=1}^{N}{\left(y_{i}-\overset{-}{y}\right)}^{2}\sum_{i=1}^{N}{\left(y_{i}-\overset{-}{y}\right)}^{2}}}, \end{array}$$

where *y*_*i*_ represents the observed flow record, *y*_*i*_′ is the alternative methods estimated flow values, $\overset{-}{y}$ and ${\overset{-}{y}}^{\prime }$ represent the average values of the corresponding variable, and *N* represents the amount of data considered. Additionally, a linear regression *y* = *α*_1_ *x* + *α*_0_ is applied for evaluating the models' performance statistically, where *y* is the dependent variable (alternative methods), *x* the independent variable (observed), *α*_1_ the slope, and *α*_0_ the intercept.

## 3. Results and Discussion

### 3.1. Processing Elements Determination

**Figure 4:**
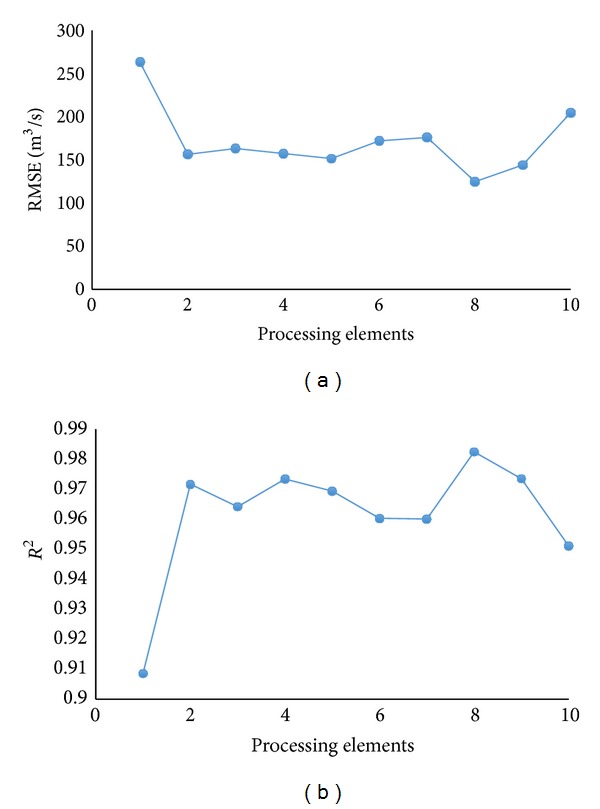
MLP accuracy under different number of processing elements.

The determination of processing elements (PE) is one of the difficult tasks in neural network models [10, 21, 23]. In addition, it is an important factor, which affects the performance of the trained network [24]. Hence, determination of PEs was the initial process of the learning procedure. The number of PEs in the hidden layer was varied between 1 and 10 for the MLP. The data set aside for testing was used to find the optimal number of PEs. In this study, the number of optimum PEs was found at 8 based on the minimum RMSE and maximum *R* ^2^ as illustrated by Figure 4.

**Figure 5:**
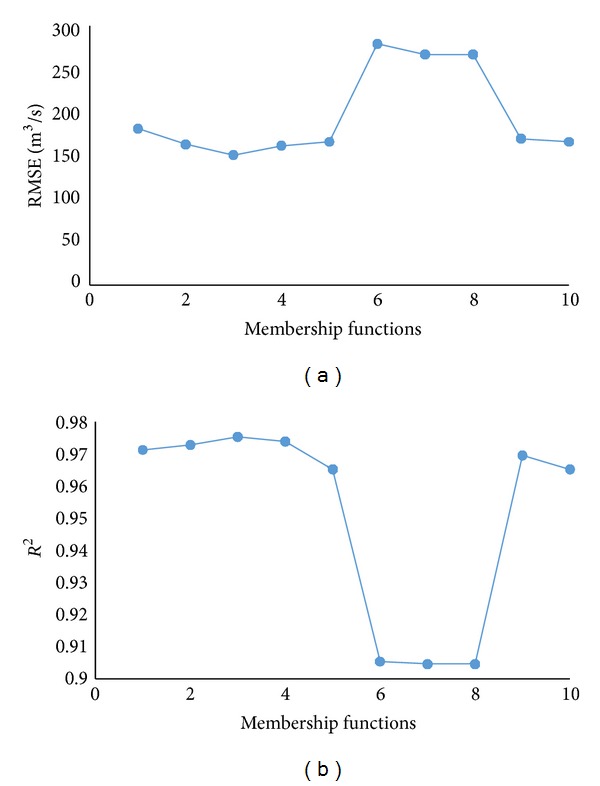
CANFISM accuracy under different number of membership functions.

In CANFISM, however, the hidden layer and the processing elements do not exist in the structure. Instead, membership functions are used. The ability of CANFISM model to achieve the performance goal depends on the internal CANFISM parameters such as the number and shape of membership functions [25]. In this study, the membership functions were varied between 1 and 10. The optimum membership function was found to be 3, with the algorithm Quickprop and the transfer function bias as proved by trial and error. This was still based on the minimum RMSE and maximum *R* ^2^ (Figure 5).

### 3.2. Comparison of the Different Models

In the present study, flow records for one station are estimated using MLP and CANFISM from three adjacent stations located in the same catchment. The data used to develop these models was obtained from annual reports of the Taiwan Water Resources Agency, Taiwan. The prediction capabilities of these models were analysed by means of comparison with observed data. A summary of the models statistical performance during training, cross validation, and testing stage is shown in Table 3. From the evaluation of these results, MLP was found to show better statistics results compared to CANFISM in the cross validation and testing stage. The RMSE of MLP for cross validation and testing stage was 382.98 m^3^/s and 150.36 m^3^/s, respectively, while that for CANFISM was 388.97 (m^3^/s) and 404.49 (m^3^/s), respectively. Moreover, the *R* ^2^ of MLP in cross validation and testing was 0.83 and 0.98, respectively, while that for CANFISM was 0.81 and 0.97, respectively. Reference [23] made similar observations in the prediction of pan evaporation that MLP model was better than CANFISM.

**Figure 6:**
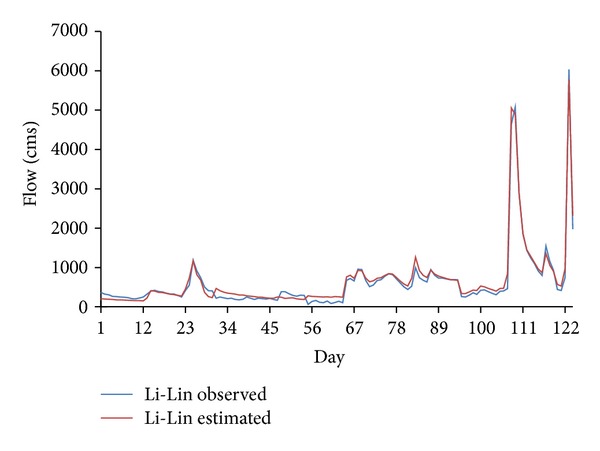
Observed and estimated flow using MLP.

**Figure 7:**
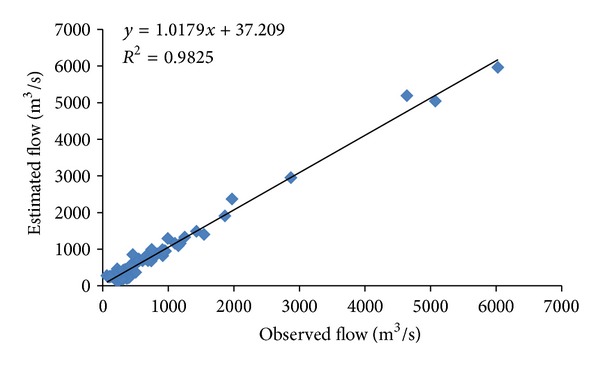
Scatter for observed and estimated flow using MLP.

**Figure 8:**
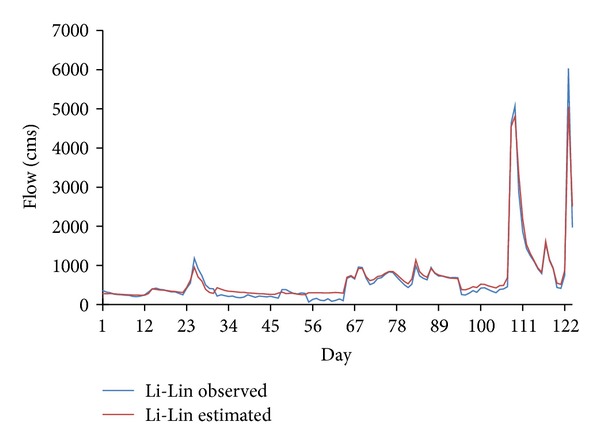
Observed and estimated flow using CANFISM.

**Figure 9:**
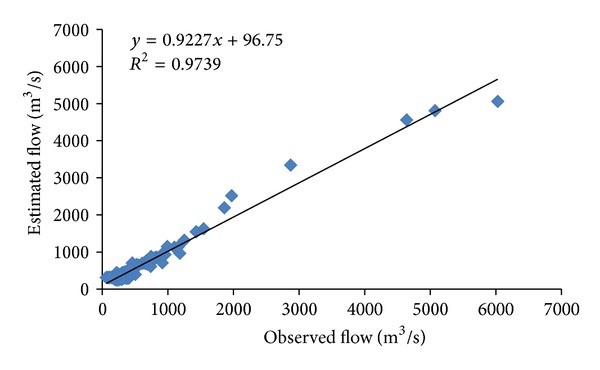
Scatter for observed and estimated flow using CANFISM.

CANFISM showed better results only in the training stage, having RMSE and *R* ^2^ of 388.97 (m^3^/s) and 0.69 compared to that of MLP, having RMSE of 401.84 (m^3^/s) and *R* ^2^ of 0.67. Figures 6 and 8 show the observed and estimated flows using MLP and CANFISM, respectively. The trends of the estimated flow are similar to the observed data, although at some places, slight differences are seen. The corresponding scatters for both MLP and CANFISM in the testing stage are shown in Figures 7 and 9. The higher accuracy attained by these models emphasizes the applicability of ANNs in estimating missing flow records.

## 4. Conclusion

Accurate estimation of missing flow records is an essential component in decision support system for efficient water management and future planning of water resources systems. The objective of the paper was to investigate the accuracy of artificial neural networks (ANN) in estimating missing flow records. The flow data of three stations was used to estimate flow data of one station. The potential of ANNs for estimating missing flow records has been demonstrated in this study with both MLP and CANFISM having higher *R* ^2^ of 0.98 and 0.97, respectively. In general, the findings of this study indicate that accurate estimations of missing flow records under the complex hydrological condition of Taiwan can be attained using MLP and CANFISM methods.

## Figures and Tables

**Table 1:** Conditions of the training performance variables for MLP.

Training variables	Assigned value
Step size	1
Momentum	0.5
Iterations	5000
Training threshold	0.001

**Table 2:** Conditions of the training performance variables for CANFISM.

Training variables	Assigned value
Membership function	Gaussian
MFs per input	3
Fuzzy model	TSK
Step size	1
Momentum	0.5
Iterations	1000
Training threshold	0.001

**Table 3:** Summary of models statistical performance.

Model	Stage	RMSE (m^3^/s)	MAE (m^3^/s)	*R* ^2^
MLP	Training	401.84	213.12	0.67
	Cross validation	382.98	170.96	0.83
	Testing	124.71	96.33	0.98

CANFISM	Training	388.97	209.28	0.69
	Cross validation	404.49	184.48	0.81
	Testing	150.36	95.99	0.97

## References

[1] Cheng C-T, Wang W-C, Xu D-M, Chau KW (2008). Optimizing hydropower reservoir operation using hybrid genetic algorithm and chaos. *Water Resources Management*.

[2] Haan CT (1977). *Statistical Models in Hydrology*.

[3] Beauchamp JJ, Downing DJ, Railsback SF (1989). Comparison of regression and time-series methods for synthesizing missing streamflow records. *Water Resources Bulletin*.

[4] Van Geer FC, Zuur AF (1997). An extension of Box-Jenkins transfer/noise models for spatial interpolation of groundwater head series. *Journal of Hydrology*.

[5] Tokar AS, Johnson PA (1999). Rainfall-runoff modeling using artificial neural networks. *Journal of Hydrologic Engineering*.

[6] Wang YM, Chen SM, Tsou I (2013). Using artificial neural network approach for modelling rainfall-runoff. *Journal of Earth System Science*.

[7] Besaw LE, Rizzo DM, Bierman PR, Hackett WR (2010). Advances in ungauged streamflow prediction using artificial neural networks. *Journal of Hydrology*.

[8] Dastorani MT, Wright NG (2004). A hydrodynamic/neural network approach for enhanced river flow prediction. *International Journal of Civil Engineering*.

[9] Szidarovszky F, Coppola EA, Long J, Hall AD, Poulton MM (2007). A hybrid artificial neural network-numerical model for ground water problems. *Ground Water*.

[10] Wang Y-M, Traore S (2009). Time-lagged recurrent network for forecasting episodic event suspended sediment load in typhoon prone area. *International Journal of Physical Sciences*.

[11] Dastorani MT, Moghadamnia A, Piri J, Rico-Ramirez M (2010). Application of ANN and ANFIS models for reconstructing missing flow data. *Environmental Monitoring and Assessment*.

[12] Wu CL, Chau KW, Li YS (2008). River stage prediction based on a distributed support vector regression. *Journal of Hydrology*.

[13] Coulibaly P, Evora ND (2007). Comparison of neural network methods for infilling missing daily weather records. *Journal of Hydrology*.

[14] Aytek A (2009). Co-active neurofuzzy inference system for evapotranspiration modeling. *Soft Computing*.

[15] El-Shafie A, Taha MR, Noureldin A (2007). A neuro-fuzzy model for inflow forecasting of the Nile river at Aswan high dam. *Water Resources Management*.

[16] Kişi Ö, Öztürk Ö (2007). Adaptive neurofuzzy computing technique for evapotranspiration estimation. *Journal of Irrigation and Drainage Engineering*.

[17] Lin J-Y, Cheng C-T, Chau K-W (2006). Using support vector machines for long-term discharge prediction. *Hydrological Sciences Journal*.

[18] Haykin S (2009). *Neural Networks and Learning Machines*.

[19] Wang Y-M, Traore S, Kerh T, Leu J-M (2011). Modelling reference evapotranspiration using feed forward backpropagation algorithm in arid regions of Africa. *Irrigation and Drainage*.

[20] Feyzolahpour M, Rajabi M, Roostaei S (2012). Estimating suspended sediment concentration using neural differential evolution (NDE), multilayer perceptron (MLP) and radial basis function (RBF) models. *International Journal of Physical Sciences*.

[21] Lin C-C (2006). Partitioning capabilities of multi-layer perceptrons on nested rectangular decision regions part I: algorithm. *WSEAS Transactions on Information Science and Applications*.

[22] Kim S, Park KB, Seo YM (2012). Estimation of Pan Evaporation using neural networks and climate based models. *Disaster Advances*.

[23] Tabari H, Talaee PH, Abghari H (2012). Utility of coactive neuro-fuzzy inference system for pan evaporation modeling in comparison with multilayer perceptron. *Meteorology and Atmospheric Physics*.

[24] Muttil N, Chau K-W (2006). Neural network and genetic programming for modelling coastal algal blooms. *International Journal of Environment and Pollution*.

[25] Heydari M, Talaee PH (2011). Prediction of flow through rockfill dams using a neuro-fuzzy computing technique. *Journal of Mathematics and Computer Science*.

